# Smoking is associated with worse outcomes of COVID-19 particularly among younger adults: a systematic review and meta-analysis

**DOI:** 10.1186/s12889-021-11579-x

**Published:** 2021-08-16

**Authors:** Roengrudee Patanavanich, Stanton A. Glantz

**Affiliations:** 1 Center for Tobacco Control Research and Education, Department of Medicine, University of California San Francisco, San Francisco, CA 94143-1390 USA; 2 Department of Community Medicine, Faculty of Medicine Ramathibodi Hospital, Mahidol University, Nakhon Pathom, Thailand

**Keywords:** COVID-19, Coronavirus, Meta-analysis, Disease progression, Odds ratio, Death, Age effect

## Abstract

**Background:**

Smoking impairs lung immune function and damages upper airways, increasing risks of contracting and severity of infectious diseases. This paper quantifies the association between smoking and COVID-19 disease progression.

**Methods:**

We searched PubMed and Embase for studies published from January 1–May 25, 2020. We included studies reporting smoking behavior of COVID-19 patients and progression of disease, including death. We used random effects meta-analysis, meta-regression and locally weighted regression and smoothing to examine relationships in the data.

**Results:**

We identified 46 peer-reviewed papers with a total of 22,939 COVID-19 patients, 5421 (23.6%) experienced disease progression and 2914 (12.7%) with a history of smoking (current and former smokers). Among those with a history of smoking, 33.5% experienced disease progression, compared with 21.9% of non-smokers. The meta-analysis confirmed an association between ever smoking and COVID-19 progression (OR 1.59, 95% CI 1.33–1.89, *p* = 0.001). Ever smoking was associated with increased risk of death from COVID-19 (OR 1.19, 95% CI 1.02–1.39, *p* = 0.003). We found no significant difference (*p* = 0.864) between the effects of ever smoking on COVID-19 disease progression between adjusted and unadjusted analyses, suggesting that smoking is an independent risk factor for COVID-19 disease progression. We also found the risk of having COVID-19 progression higher among younger adults (*p* = 0.001), with the effect most pronounced among younger adults under about 45 years old.

**Conclusions:**

Smoking is an independent risk for having progression of COVID-19, including mortality. The effects seem to be higher among young people. Smoking prevention and cessation should remain a priority for the public, physicians, and public health professionals during the COVID-19 pandemic.

**Supplementary Information:**

The online version contains supplementary material available at 10.1186/s12889-021-11579-x.

## Background

Coronavirus disease 2019 (COVID-19) first occurred in Wuhan, China in December 2019 and has spread rapidly around the world. As of April 2021, the virus had infected over 150 million people and caused more than 3 million deaths [1]. Old people and those with pre-existing medical conditions including respiratory disease, hypertension, diabetes, cardiovascular disease, and cancer are more vulnerable to becoming critically ill when infected [2].

Smoking may enhance the risk of COVID-19 by its biological effects and behaviors of smokers. Smoking impairs lung function and pulmonary immune function, compromising the body’s defense mechanisms against infections [3]. Smoking is also a well-established risk factor for chronic diseases that are linked to more severe COVID-19. The World Health Organization (WHO) has advised the public that smoking could increase the risk of contracting COVID-19 because the behavior of smokers involves contact of fingers with the lips and removal of the protective face masks to smoke [4].

Our recent meta-analysis of the 19 peer-reviewed papers found that smokers have double the odds of COVID-19 progression risk [5]. Some people argue that the association between underlying health conditions and risk factors such as smoking to the severity of COVID-19 is still unclear due to inadequate adjustment of confounding factors [6]. In addition, it is unclear whether the association between smoking and severity of COVID-19 varies by age. This paper updates and extends our previous meta-analysis [5] of 19 studies to add 27 additional studies, including 6 that provided adjusted odds ratios and compared the association between smoking and COVID-19 disease progression between unadjusted analyses with adjusted analyses to examine whether smoking is an independent risk factor. We also assessed the effect of age of patients and conducted a sub-analysis for the risk of smoking on the mortality of COVID-19.

## Methods

This study followed the Preferred Reporting in Systematic Reviews and Meta-Analyses (PRISMA) guidelines and is registered with PROSPERO (CRD42020186864).

### Data source and search strateg*y*

We conducted a systematic search using PubMed and Embase on May 25, 2020, with the search term: “((smoking) OR (characteristics) OR (risk factors) OR (retrospective*) OR (outcomes) OR (smoker*)) AND ((COVID-19) OR (COVID) OR (coronavirus) OR (sars cov-2) OR (sars cov 2))” for studies published between January 1, 2020 and May 25, 2020. A total of 2600 studies were retrieved through PubMed and 1962 studies through Embase.

### Eligibility criteria

Eligible studies included published peer-reviewed observational studies, retrospective cohort studies, prospective cohort studies, cross-sectional studies, case series, and case reports that reported demographic characteristics, comorbidities specifically smoking status, clinical manifestations, and clinical or disease outcomes of COVID-19 patients on disease progression of COVID-19 to more severe or critical conditions or death. We included both inpatient and outpatient settings. We excluded studies that did not report smoking status and outcomes, studies of children, studies that included other coronavirus infection and not specifically to COVID-19, studies that the number of smokers was zero or omitted, and studies in which all patients had the same outcome. There were no language restrictions.

### Study selection and data extraction

One author (RP) extracted information for each study, screened the abstract or the full text, with questions resolved through discussion among both authors (Fig. A1).

The exposure group for our analysis were those who had a history of smoking (current smokers and former smokers) and unexposed group was never smokers, non-smokers, or not having a smoking history. Outcomes were progression of COVID-19 to more severe or critical conditions or death. Definitions of smoking status and disease progression for each study are shown in Table A1.

### Quality assessment

We evaluated the quality of studies using a modification of the ACROBAT-NRSI [7] tool on 5 domains: study population, exposure measurement, outcome assessment, measurement of confounders, and adequate follow-up. Each one of these domains was scored from 0 (low risk of bias) to 2 (high risk of bias) and the average score of each study was computed and discussed among both authors (Additional file and Table A2). Studies with the average score higher than 1 were considered high risk and excluded in a sensitivity analysis.

### Statistical analyses

Our meta-analyses were based on unadjusted odds ratios (OR) that were either reported in the studies or computed unadjusted OR and 95% confidence interval (CI) using the number of smokers (current and former) and never smokers with and without disease progression. We also did a sensitivity analysis to determine the results changed when the 5 studies with high risk of bias were excluded.

We performed subgroup analyses of (1) the studies that reported association of smoking on COVID-19 mortality and (2) the association of COVID-19 disease progression between current smokers and never smokers (i.e., excluding former smokers), and former smokers and never smokers using the studies that reported whether the patient was a current, former, or never smoker (as separate categories).

We also computed the pooled adjusted OR using the studies that reported adjusted OR and 95% CI and compared it with the pooled unadjusted OR.

The results of the included studies were pooled with random-effect models using the Stata version 14.0 *metan* command and *metabias* command with Egger’s test for the presence of publication bias. We used *metareg* command (with dummy variables to account for the pairing of adjusted and unadjusted ORs) to determine whether the adjustment of OR affected the results. We used locally weighted regression and smoothing because both visual inspection and analysis of residuals using a preliminary linear regression indicated a nonlinear relationship between odds of disease progression and mean or median age reported in each study. The *lowess* command was used to generate a nonparametric fit estimate. We also tested for a trend using *metareg* command with mean age of each study as a continuous variable.

## Results

### Study characteristics

From the total of 4562 studies we found from our search, 237 studies were considered retrospective cohorts, prospective cohorts, or case series that provided clinical and demographic characteristics of COVID-19 patients. From the 237 studies, 83 studies reported smoking status of the patients, but only 47 studies [8–54] reported smoking status and disease progression of COVID-19 that met our inclusion and exclusion criteria. One study [31] was later retracted so that the final analysis is based on 46 studies [8–30, 32–54]. (Fig. A1).

Of the 46 studies (Table A1), 33 [11–14, 18–22, 24, 27–30, 32, 36, 38–54] were from China, 8 [9, 10, 15, 17, 23, 26, 34, 35] from the US, 3 [16, 33, 37] from Italy, 1 [8] from the UK, and 1 [25] from South Korea.

Seven studies [10, 12, 18, 27, 35, 37, 50] assessed whether the patient was a current, former, or never smoker (as separate categories), 15 [13, 15, 17, 21, 32, 40–44, 46, 47, 51, 52, 54] studies assessed whether the patient was a “current smoker”, 24 [8, 9, 11, 14, 16, 19, 20, 22–26, 28–30, 33, 34, 36, 38, 39, 45, 48, 49, 53] studies assessed whether the patient had a “history of smoking” (current and former). Forty-five studies [8, 9, 11–30, 32–54] reported the mean or median ages of patients.

Clinical outcome was defined as death in 7 studies [12, 16, 26, 33, 34, 45, 54], intensive care unit (ICU) admission or requirement of mechanical ventilation in 6 studies [8, 10, 15, 17, 21, 23], prolonged viral shredding in 2 studies [43, 44], severe or critical (respiratory distress with respiratory rate ≥ 30/min, or oxygen saturation ≤ 93% at rest, or oxygenation index ≤300 mmHg, based on the diagnostic and treatment guideline for SARS-CoV-2 issued by Chinese National Health Committee [55] or the American Thoracic Society guidelines [56] for community acquired pneumonia) in 18 studies, the primary composite end point (ICU admission, the use of mechanical ventilation, or death) in 2 studies [18, 47], abnormal chest imaging in 1 study [52], acute cardiac injury in 1 study [19], progression of disease to more severe status (including increasing oxygen supplement, pneumonia exacerbation, transferred to ICU, and sepsis) in 10 studies [13, 20, 24, 25, 30, 32, 34, 37, 38, 48]. Forty-five studies [8–30, 32, 33, 35–54] reported the number of smokers by clinical outcomes.

There were 5 studies [8, 13, 15, 33, 39] with high risk of bias scores (Table A2).

### Ever smoking and COVID-19 disease progression

**Fig. 1:**
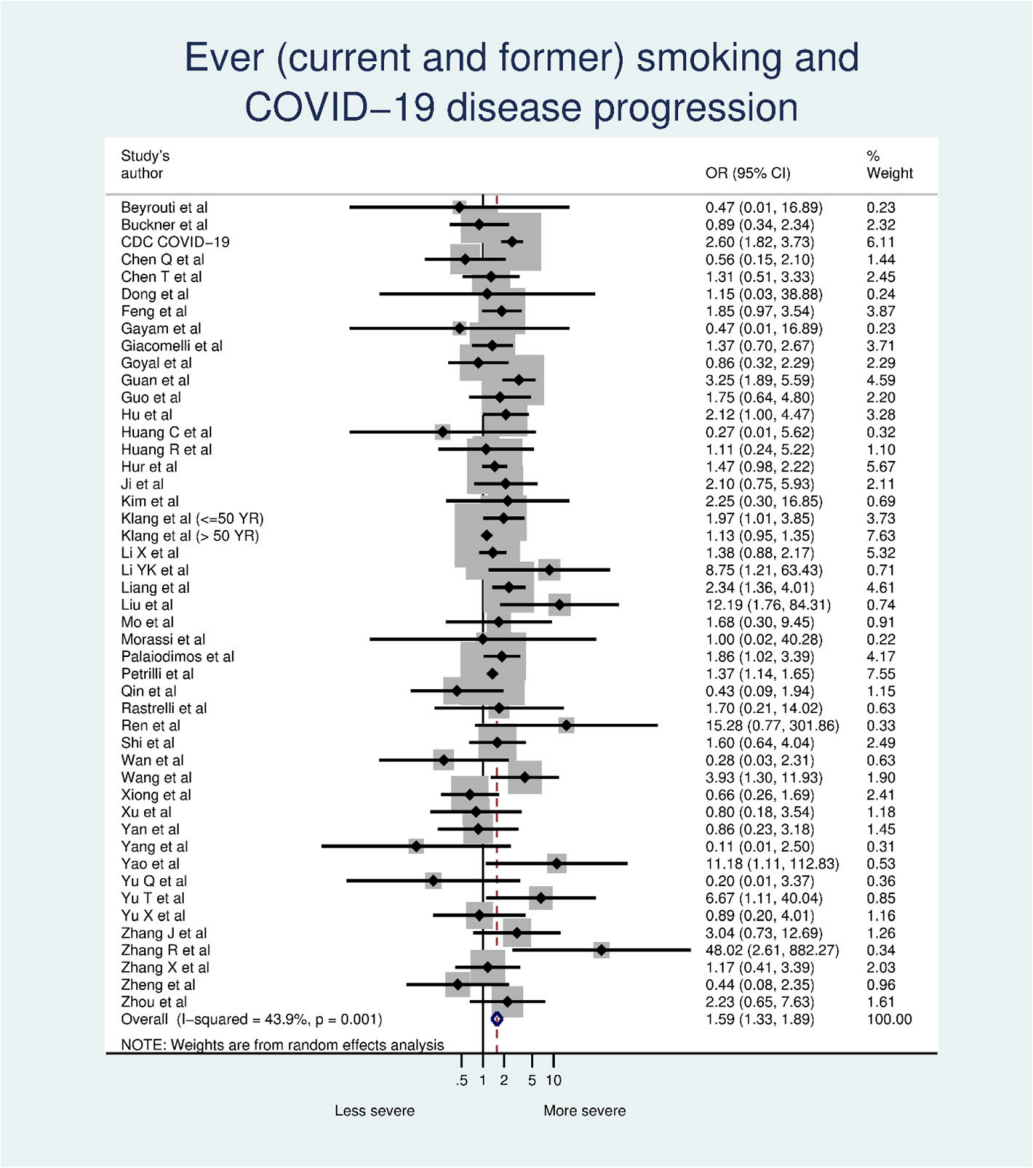
Ever (current and former) smoking and COVID-19 disease progression

A total of 22,939 COVID-19 patients are included in our meta-analysis, 5421 of whom (23.6%) experienced disease progression and 2914 (12.7%) with a history of smoking (current and former smokers). Among those with a history of smoking, 33.5% experienced disease progression, compared with 21.9% of non-smokers. The meta-analysis showed an association between ever smoking and COVID-19 progression (OR 1.59, 95% CI 1.33–1.89, *p* = 0.001) (Fig. 1). There was statistically significant moderate heterogeneity among the studies (I^2^ = 43.9%, p = 0.001) and no evidence of publication bias (*p* = 0.387).

Smoking prevalence (ever smoking) was significantly higher among patients with disease progression than those without disease progression (16.1% vs. 11.6%,; *p* = 0.023 by paired t-test).

### Ever smoking and COVID-19 mortality

Seven studies [12, 16, 26, 33, 34, 45, 54] (*n* = 4362) reported death as the outcome of COVID-19 patients. The meta-analysis showed an association between ever smoking and COVID-19 mortality (OR 1.19, 95% CI 1.02–1.39, *p* = 0.003; Fig. A3). There was no evidence of heterogeneity among the studies (I^2^ = 0%, *p* = 0.548) and no evidence of publication bias (*p* = 0.662).

### Studies of current vs. never smokers and former vs. never smokers

The 7 studies [10, 12, 18, 27, 35, 37, 50] that reported current, former vs. never smokers yielded an elevated point estimate for the effect of current smokers on COVID-19 progression (OR 1.35, 95% CI 0.83–2.22, *p* = 0.230; Fig. A4), but it did not reach conventional statistical significance. There was statistically significant moderate heterogeneity (I^2^ = 56.3%, *p* = 0.033) and no evidence of publication bias (*p* = 0.368). Former smokers were more likely to be at increased risk of COVID-19 disease progression compared with never smokers (OR = 2.27, 95% CI = 1.34–3.85, *p* = 0.002). There was statistically significant high heterogeneity (I^2^ = 75.6%, *p* = 0.001) and no evidence of publication bias (*p* = 0.387).

### Unadjusted vs. adjusted analyses

Six studies [20, 26, 30, 34, 35, 48] reported adjusted ORs. The point estimate for the adjusted ORs (OR 1.95, 95% CI 1.12–3.41, *p* = 0.018; Fig. A5, top) was higher than the point estimate for unadjusted ORs (OR 1.60, 95% CI 1.11–2.33, *p* = 0.013; Fig. A5, bottom), but this difference was not significant (*p* = 0.864). For the adjusted ORs, the heterogeneity among the studies was high and statistically significant (I^2^ = 80.2%, *p* = 0.001) with evidence of publication bias (*p* = 0.003). For the unadjusted ORs, the heterogeneity among the studies was moderate and statistically significant (I^2^ = 65.7%, *p* = 0.008) with evidence of publication bias (*p* = 0.002).

### Association between ever smoking and COVID-19 disease progression by age

**Fig. 2:**
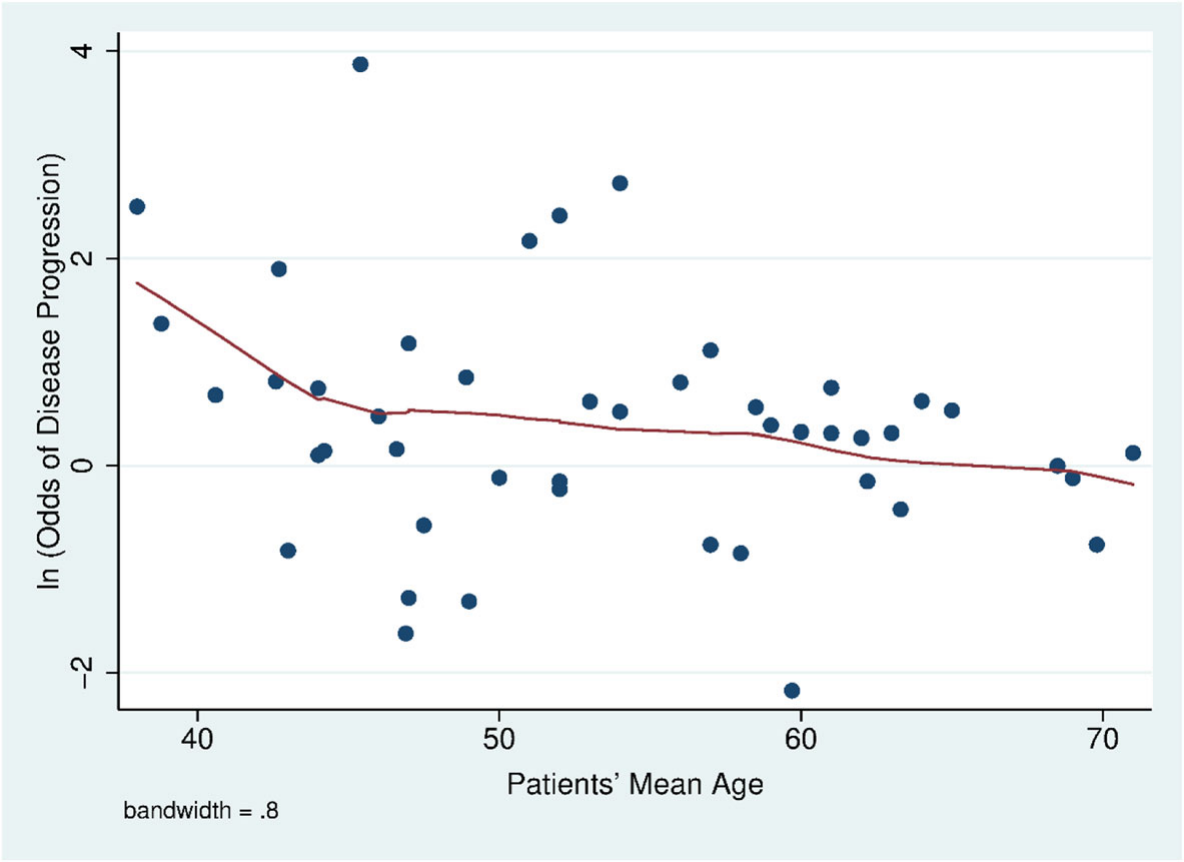
As the mean or median age of patients in a study falls the odds of COVID-19 progression increases. Solid line fit using a locally weighted regession and smoothing

The odds of COVID-19 disease progression between smokers and non-smokers dropped as the patients’ mean age increased across studies, with the drop most pronounced for studies where the mean age was less than about 45 years old (Fig. 2). A meta-regression of the odds of COVID-19 disease progression between smokers and non-smokers and the patients’ mean age showed that each the odds of disease progression dropped statistically significantly by a factor of 0.78 (95% CI 0.69–0.89, *p* = 0.001) per 10 years.

### Sensitivity analysis

Dropping the 5 studies [8, 13, 15, 33, 39] with high risk of bias scores had little effect on the odds of COVID-19 disease progression (OR 1.60, 95% CI 1.33–1.92, *p* = 0.001; Fig. A2). The heterogeneity among the studies was moderate and statistically significant (I^2^ = 49.5%, p = 0.001) and there was no evidence of publication bias (*p* = 0.278).

## Discussion

With more than twice as many studies available compared to our earlier meta-analysis [5], smoking remains a risk factor for COVID-19 disease progression, with smokers having 1.59 times the odds of progression in COVID-19 severity than non-smokers (Fig. 1). The risk of smoking on COVID-19 disease progression was not changed significantly by adjusting for confounders, which suggests that smoking is an independent risk of COVID-19 progression. We also find that smokers are at increased risk of death from COVID-19 (Fig. A3). These findings are not surprising because the well-established evidence that smoking is associated with a higher risk of viral infection [3]. In the past pandemics such as influenza [3] and Cov-MERS [57] smoking is also among leading risk factors for worse outcomes.

Although the studies included in our paper (published as of May 25, 2020) reflect the first wave of COVID-19 pandemic, our results (OR 1.59, 95% CI 1.33–1.89, *p* = 0.001) are consistent with a meta-analyses published after our paper was submitted in September 2020 based on 109 studies from when the pandemic started [58] to February 2021 (1.55, 95% CI 1.41–1.71) [59]. Our finding of a higher OR for former than current smokers is also consistent with other analyses published after our paper was submitted [58, 59].

Younger smokers appear to have a higher risk of COVID-19 disease progression than older smokers (Fig. 2). A recent study also found that younger adults are more medically vulnerable to severe COVID-19 illness if they are smokers [60]. The greater effect of smoking among young people is particularly important because in the U.S., almost 40% of COVID-19 patients are aged 18–44 years [61], and in China, 44% of COVID-19 patients are adults aged 20–49 years [62]. Even so, younger adults tend to perceiving lesser infection-fatality risks of COVID-19 [63] so that they are less likely to protect themselves from the infection. Our finding is consistent with a recent meta-analysis study [64] which concluded that age was negatively significantly associated with the effect of smoking on COVID-19 disease severity.

While there is not yet direct peer reviewed evidence of the effect of e-cigarette use on COVID-19 risk, the fact that e-cigarettes have similar adverse effects on pulmonary immune function [65] combined with the fact that e-cigarette use is concentrated among younger people, raises concerns and points to the need to collect data on e-cigarette use and COVID-19 risk.

Some have argued that smoking has a protective effect against COVID-19 because of the low smoking prevalence of reported among COVID patients [66–68]. This is not new. There were also rumors that smoking protected patients from developing Cov-SARS during the 2003 pandemic [69]. However, a case-control study of 447 patients showed that smoking did not protect patients from contracting Cov-SARS after adjusting for confounding by age, gender, contact history, and occupation [69].

Reported smoking prevalence in the 33 studies in China ranged from 1.4 to 29.8% (median = 7.3%), which was substantially lower than 27.7% (52.1% for men and 2.7% for women) smoking prevalence in 2015 [70]. Four studies [10, 15, 17, 35] in the U.S. that reported the smoking prevalence among current smokers ranged from 1.3 to 33.3% (median = 5.2%), which was also lower than 13.7% (15.6% for men and 12.0% for women) smoking prevalence in 2018 [71]. The other 4 studies [9, 23, 26, 34] in the U.S. reported the ever-smoking prevalence ranged from 13.3–33.5%, which was also lower than 41.9% (47.2% for men and 37.3% for women) in 2017 [72]. One study [37] in Italy reported the smoking prevalence among current smokers of 3.2%, which was also substantial lower than 21.1% (26% for men and 17.2% for women) in 2016 [73]. The remaining studies that reported the ever-smoking prevalence (2 studies [16, 33] in Italy, 1 study [8] in UK and 1 study [25] in South Korea) were also lower than the countries’ rates (Italy: 16.7–30% vs.43.9% (50% for men and 38.3% for women) in 2010 [74]; UK: 16.7% vs. 40.2 (44.3% for men and 36.5% for women) in 2018 [75]; South Korea: 18.5% vs.39.1% (81.6% for men and 6.9% for women) in 2015 [76]). These low levels of reported smoking among COVID-19 patients may reflect the difficulty of obtaining accurate smoking histories among seriously ill patients, especially when most medical facilities are operating at or above normal capacity. Despite the fact that the reported levels of smoking have been below population prevalences; however, the reported smoking prevalence among people with worse outcomes was significantly higher than those with less severe outcomes (16.1% vs. 11.6%, *p* = 0.023).

### Limitations

The studies used a variety of clinical definitions of disease progression and smoking status (Table A1). (This is a common practical problem when conducting meta-analyses.) The varying definitions of disease progression include severity of disease based on clinical manifestations, development to more severe conditions, increasing oxygen supplements, prolonged viral shredding, organ injuries, ICU admission, and death. These varying definitions likely introduced increased variance in the pooled risk estimates and probably accounts for at least some of the heterogeneity between studies that was observed.

However, smoking was significantly associated with death – a clearly defined endpoint – in the 7 studies [12, 16, 26, 33, 34, 45, 54] that used this endpoint.

Most studies reported smoking status as having a smoking history, often without clearly stating how they categorized former smokers. Of the 46 studies we reviewed, only 7 [10, 12, 18, 27, 35, 37, 50] reported all three smoking categories (current, former, and never smokers). A meta-analysis of these studies found that current smoking was associated with a similar increase in the point estimate for the odds of disease progression (OR 1.35, 95% CI 0.83–2.22; Fig. A4) as the other studies (OR 1.54, 95% CI 1.24–1.91), but the odds for current smoking did not reach conventional statistical significance (*p* = 0.202).

Studies that only describe patient smoking history as “smoking history” or “history of smoking” do not provide enough information to analyze smoking as a risk factor given the fact that time since quitting could have significant influence on the patient’s outcomes.

When estimating adjusted ORs, it is important to have an appropriately specified model. The studies that reported adjusted odds ratios accounted for a variety of covariates (Table A1), which means that the resulting ORs are not strictly comparable [77]. (This is another commonly-encountered problem in conducing meta-analyses.)

All these limitations add to misclassification errors, which tend to bias results toward the null, suggest that this analysis underestimates the risk of smoking in terms of increasing COVID-19 severity.

The effects of smoking on COVID-19 disease progression by age reported in our paper is limited to the mean or median age in the studies. Individual level data on smoking, age, e-cigarette use, demographics and other risk factors are needed to perform a more sophisticated analysis. In addition, most of the studies were retrospective cohorts or case series, there might be recall bias, and could not conclude a causal relationship. Most of the meta-analyses in this study had moderate and statistically significant heterogeneity; the reliability of the meta-analyses might be compromised.

## Conclusions

Smoking is an independent risk associated with severe progression of COVID-19, including mortality. The effects seem to be larger among younger adults. Smoking prevention and cessation should remain a priority for the public, physicians, and public health professionals during the COVID-19 pandemic.

## Supplementary Information

**Additional file 1.** Risk of Bias Assessment Method. Table A1. Summary of Studies. Table A2. Risk of Bias of Studies (2 = high risk, 1 = intermediate risk, 0 = low risk). Fig. A1. PRISMA diagram. Fig. A2. Ever (current and former) smoking and COVID-19 disease progression, dropping 5. studies with high risk of bias scores. Fig. A3. Ever (current and former) smoking and COVID-19 mortality. Fig. A4. Current smokers vs. never smokers and COVID-19 disease progression. Fig. A5. Ever (current and former) smoking and disease progression in models that adjusted (top) and did not adjust (bottom) ORs for confounding variables.

## Data Availability

All data used in this meta-analysis are freely and publicly available from the cited papers used in the analysis; the full citations are in the reference list.

## Abbreviations

COVID-19: Coronavirus disease 2019
CI: Confidence interval
OR: Odds ratio
ICU: Intensive care unit
