# Supplementary material for: Smoking is associated with worse outcomes of COVID-19 particularly among younger adults: a systematic review and meta-analysis
Source: BMC Public Health. 2021 Aug 16;21:1554. doi: 10.1186/s12889-021-11579-x (PMC8366155; doi:10.1186/s12889-021-11579-x)
Supplement: Supplementary file 1 — Additional file 1. Risk of Bias Assessment Method. Table A1. Summary of Studies. Table A2. Risk of Bias of Studies (2 = high risk, 1 = intermediate risk, 0 = low risk). Fig. A1. PRISMA diagram. Fig. A2. Ever (current and former) smoking and COVID-19 disease progression, dropping 5. studies with high risk of bias scores. Fig. A3. Ever (current and former) smoking and COVID-19 mortality. Fig. A4. Current smokers vs. never smokers and COVID-19 disease progression. Fig. A5. Ever (current and former) smoking and disease progression in models that adjusted (top) and did not adjust (bottom) ORs for confounding variables. [file 12889_2021_11579_MOESM1_ESM.pdf]

Smoking is associated with worse outcomes of COVID-19 particularly among younger adults: A systematic review and meta-analysis

ADDITIONAL FILE

Roengrudee Patanavanich MD LLM PhD
Stanton A. Glantz PhD

Risk of Bias Assessment

We evaluated the quality of studies using a modification of the ACROBAT-NRSI[7] tool on 5 domains: study population, exposure measurement, outcome assessment, measurement of confounders, and adequate follow-up. Each one of these domains was scored 0 for low risk of bias, 1 for moderate risk of bias, and 2 for high risk of bias and the average score of each study was computed and discussed among both authors. Studies with the average score higher than 1 were considered high risk and excluded in a sensitivity analysis.

Study population

- Low risk: studies included at least 50 patients
- Moderate risk: studies included at least 20 patients
- High risk: studies included less than 20 patients

Exposure measurement

- Low risk: studies reported 3 categories of smoking status: current, former, and never smokers
- Moderate risk: studies reported 2 categories of smoking status
- High risk: studies reported only 1 category of smoking status

Outcome assessment

- Low risk: studies reported a clear definition of outcomes
- High risk: studies did not report specific outcomes

Measurement of confounders

- Low risk: adjustment for confounders
- High risk: unadjusted analyses

Adequate follow-up

- Low risk: identify duration of data collection
- High risk: duration of data collection is unclear

References

References are the same as cited in the main text.

Table A1 Summary of Studies													
**Author**	**Setting**	**Study type**	**Time of data collection**	**Population**	**Definition of disease progression**	**Smoking status**	**Odds of disease progression (95%CI)**		**Adjustment**	**Smoking prevalence**			**Patients' mean age**
							**Unadjusted**	**Adjusted**		**Overall**	**More severe group**	**Less severe group**	
Beyrouti et al [8]	UK National Hospital for Neurology and Neurosurgery, Queen Square, London	Case series	1 Apr 2020-16 Apr 2020	6 confirmed COVID-19 patients with acute ischemic stroke	ICU admission	Smoking	0.47 (0.01, 16.89)	N/A	N/A	16.7%	N/A	25.0%	70
Buckner et al[9]	US University of Washington affiliated hospitals	Retrospective	2 Mar 2020-26 Mar 2020	105 hospitalized patients with laboratory confirmed COVID-19	A composite endpoint of admission to an intensive care unit (ICU) or death	Smoking (ever)	0.89 (0.34, 2.35)	N/A	N/A	25.6%	24.4%	26.7%	69
CDC COVID-19 Response Team [10]	US	Retrospective	12 Feb 2020-28 Mar 2020	7,162 confirmed COVID-19 patients with completed information*	ICU admission	Current and former smoker	Current smoker: 0.86 (0.35-2.13) Former smoker: 3.76 (2.53-5.59) Current/former: 2.60 (1.82-3.73)	N/A	N/A	Current smoker: 1.3% Former smoker: 2.3% Current/former: 3.6%	Current smoker: 0.5% Former smoker: 3.1% Current/former: 3.6%	Current smoker: 1.3% Former smoker: 2.0% Current/former: 3.4%	N/A
Chen Q et al [11]	China Taizhou Public Health Medical Center, Zhejiang	Retrospective	1 Jan 2020- 11 Mar 2020	145 hospitalized patients with laboratory confirmed COVID-19	Respiratory distress with respiratory rate ≥30/min, or oxygen saturation ≤93% at rest, or oxygenation index ≤ 300 mmHg	Smoking history	0.56 (0.15-2.10)	N/A	N/A	10.3%	7.0%	11.8%	48
Chen T et al [12]	China Tongji Hospital	Retrospective	13 Jan 2020-13 Feb 2020	799 moderately to severely ill or critically ill patients with confirmed covid-19	Death	Current and former smoker	Current smoker: 2.03 (0.63-6.58) Former smoker: 0.58 (0.11-3.05) Current/former: 1.31 (0.51-3.33)	N/A	N/A	Current smoker: 4.4% Former smoker: 2.6% Current/former: 6.9%	Current smoker: 6.2% Former smoker: 1.8% Current/former: 8.0%	Current smoker: 3.1% Former smoker: 3.1% Current/former: 6.2%	62

Table A1 Summary of Studies													
**Author**	**Setting**	**Study type**	**Time of data collection**	**Population**	**Definition of disease progression**	**Smoking status**	**Odds of disease progression (95%CI)**		**Adjustment**	**Smoking prevalence**			**Patients' mean age**
							**Unadjusted**	**Adjusted**		**Overall**	**More severe group**	**Less severe group**	
Dong et al [13]	China Zhongnan Hospital of Wuhan University, No. 7 hospital of Wuhan, and Wuhan Children's Hospital	Case series	20 Jan 2020-3 Mar 2020	9 adults hospitalized patients with laboratory confirmed COVID-19**	Bilateral pneumonia, respiratory distress, required mechanical ventilation, ICU care, or hospitalized > 10 days.	Current smoker	1.15 (0.03-38.88)	N/A	N/A	11.1%	14.3%	0.0%	44
Feng et al [14]	Jinyintan Hospital in Wuhan, Shanghai Public Health Clinical Center in Shanghai and Tongling People's Hospital in Anhui Province	Retrospective	1 Jan 2020-15 Feb 2020	476 hospitalized patients with laboratory confirmed COVID-19	Severe (respiratory distress with respiratory rate ≥30/min, or oxygen saturation ≤93% at rest, or oxygenation index ≤ 300 mmHg), or critical (respiratory failure, shock, or organ dysfunction)	Smoking	1.85 (0.97-3.54)	N/A	N/A	9.7%	14.0%	8.1%	53
Gayam et al [15]	Interfaith Medical Center, Brooklyn, New York	Case series	1 Mar 2020-15 April 2020	6 confirmed hospitalized COVID-19 patients coinfected with Mycoplasma pneumonae	Required intensive mechanical ventilator	Current smoker	0.47 (0.01-16.89)	N/A	N/A	33.3%	0.0%	40.0%	57
Giacomelliet al [16]	Infectious Diseases and Intensive Care units of Luigi Sacco Hospital, Milan	Prospective cohort	21 Feb 2020-19 Mar 2020	233 hospitalized patients with laboratory confirmed COVID-19	Death	History of smoking	1.37 (0.70-2.68)	N/A	N/A	30.0%	35.4%	28.6%	61

Table A1 Summary of Studies

Author	Setting	Study type	Time of data collection	Population	Definition of disease progression	Smoking status	Odds of disease progression (95%CI)		Adjustment	Smoking prevalence			Patients' mean age
							Unadjusted	Adjusted		Overall	More severe group	Less severe group	
Goyal et al [17]	US 862-bed quaternary referral center and an affiliated 180-bed nonteaching community hospital in Manhattan, New York	Retrospective	3 Mar 2020-27 Mar 2020	393 hospitalized patients with COVID-19 diagnosis	Required intensive mechanical ventilator	Current smoker	0.86 (0.32-2.29)	N/A	N/A	5.1%	4.6%	5.3%	62
Guan et al [18]	552 hospitals in 30 provinces, autonomous regions, and municipalities	Retrospective	11 Dec 2019-29 Jan 2020	1,099 patients (both hospitalized and outpatient) with laboratory confirmed COVID-19	ICU admission, the use of mechanical ventilation, or death	Current, former and never	Current smoker: 2.84 (1.57-5.14) Former smoker: 6.27 (2.20-17.90) Current/former: 3.25 (1.89-5.59)	N/A	N/A	Current smoker: 12.6% Former smoker: 1.9% Current/former: 14.6%	Current smoker: 25.8% Former smoker: 7.6% Current/former: 33.3%	Current smoker: 11.8% Former smoker: 1.6% Current/former: 13.3%	47
Guo et al [19]	Seventh Hospital of Wuhan City	Retrospective	23 Jan 2020-23 Feb 2020	187 hospitalized patients with laboratory confirmed COVID-19	Acute myocardial injury	Smoking	1.75 (0.64-4.80)	N/A	N/A	9.6%	13.5%	8.1%	59

Table A1 Summary of Studies													
**Author**	**Setting**	**Study type**	**Time of data collection**	**Population**	**Definition of disease progression**	**Smoking status**	**Odds of disease progression (95%CI)**		**Adjustment**	**Smoking prevalence**			**Patients' mean age**
							**Unadjusted**	**Adjusted**		**Overall**	**More severe group**	**Less severe group**	
Hu et al [20]	China Tianyou Hospital in Wuhan	Retrospective	8 Jan 2020- 20 Feb 2020	323 hospitalized patients with COVID-19 diagnosis	Death, progression from non-severe to severe (patients showed features of nonsevere patients plus respiratory distress with RR ≥30 breaths/min, oxygen saturation ≤ 93%, arterial partial pressure of oxygen/oxygen concentration ≤ 300 mmHg) or critical (patients showed respiratory failure requiring ventilatory support, as well as shock and organ dysfunction requiring intensive care) or severe to critical status, and/or maintenance of severe or critical status	Smoking history	2.12 (1.00-4.47)	3.46 (1.18-10.17)	Age, hypnotics, diagnosis at critical status, diabetes, hypertensive troponin I, white blood cell count, neutrophil count	11.8%	15.1%	7.9%	61
Huang C et al [21]	China Jin Yin-tan Hospital in Wuhan	Retrospective	16 Dec 2019-2 Jan 2020	41 hospitalized patients with laboratory confirmed COVID-19	ICU admission	Current smoking	0.27 (0.01-5.62)	N/A	N/A	7.3%	0.0%	10.7%	49
Huang R et al [22]	China 8 designated hospitals in 8 cities of Jiangsu Province	Retrospective	22 Jan 2020- 10 Feb 2020	202 hospitalized patients with laboratory confirmed COVID-19	Severe (respiratory distress with respiratory rate ≥30/min, or oxygen saturation ≤93% at rest, or oxygenation index ≤ 300 mmHg), or critical (respiratory failure, shock, or organ dysfunction)	Smoking history	1.12 (0.24, 5.22)	N/A	N/A	7.9%	8.7%	7.8%	44

Table A1 Summary of Studies													
**Author**	**Setting**	**Study type**	**Time of data collection**	**Population**	**Definition of disease progression**	**Smoking status**	**Odds of disease progression (95%CI)**		**Adjustment**	**Smoking prevalence**			**Patients' mean age**
							**Unadjusted**	**Adjusted**		**Overall**	**More severe group**	**Less severe group**	
Hur et al [23]	US<br>10 hospitals in the Chicago metropolitan area	Retrospective	1 Mar 2020- 8 April 2020	486 hospitalized patients with laboratory confirmed COVID-19	Intubation and prolonged intubation for acute respiratory failure	Current/former smoker	1.47 (0.98-2.22)	N/A	N/A	33.5%	39.9%	31.0%	59
Ji et al [24]	China<br>Fuyang Second People's Hospital and the Fifth Medical Center of Chinese PLA General Hospital	Retrospective	20 Jan 2020-22 Feb 2020	208 hospitalized patients with laboratory confirmed COVID-19	Progression to respiratory distress with respiratory rate ≥30/min, or oxygen saturation ≤93% at rest, or oxygenation index ≤ 300 mmHg, or requirement of mechanical ventilator	Smoke	2.10 (0.75-5.93)	N/A	N/A	9.1%	15.0%	7.7%	44
Kim et al [25]	Korea	Retrospective	19 Jan 2020-17 Feb 2020	28 confirmed COVID-10 cases nationwide	Required oxygen supplement	Smoking	2.25 (0.30-16.85)	N/A	N/A	21.4%	33.3%	18.2%	43
Klang et al [26]	US<br>5 hospital campuses of the Mount Sinai hospital, New York	Retrospective	1 Mar 2020- 17 May 2020	3,406 hospitalized patients with laboratory confirmed COVID-19	Death	Smoking	age ≤50: 1.97 (1.01-3.85)<br>age >50: 1.13 (0.95-1.35)	age ≤50: 1.7 (0.8-1.2)<br>age >50: 1.0 (0.8-1.2)	Age, sex, coronary arterial disease, congestive heart failure, hypertension, diabetes, hyperlipidemia, chronic kidney disease, cancer, intubation, BMI, and race	age ≤50: 13.3%<br>age >50: 25.3%	age ≤50: 21.7%<br>age >50: 26.8%	age ≤50: 12.3%<br>age >50: 24.4%	age ≤50: 41<br>age >50: 71

Table A1 Summary of Studies

Author	Setting	Study type	Time of data collection	Population	Definition of disease progression	Smoking status	Odds of disease progression (95%CI)		Adjustment	Smoking prevalence			Patients' mean age
							Unadjusted	Adjusted		Overall	More severe group	Less severe group	
Li X et al[27]	China Tongji Hospital	Retrospective	26 Jan 2020-5 Feb 2020	548 hospitalized patients with laboratory confirmed COVID-19	Respiratory distress with respiratory rate ≥30/min, or breaths per minute, PaO2/FIO2 ratio lower than 250, multilobar infiltrates confusion, or disorientation, or respiratory failure, or required mechanical ventilation, or septic shock	Current, former and never	Current smoker: 0.87 (0.46-1.66) Former smoker: 2.04 (1.12-3.73) Current/former: 1.38 (0.88-2.17)	N/A	N/A	Current smoker: 7.5% Former smoker: 9.4% Current/former: 16.9%	Current smoker: 6.8% Former smoker: 12.5% Current/former :19.2%	Current smoker: 8.2% Former smoker: 6.5% Current/former: 14.7%	60
Li YK et al[28]	China Department of Thoracic Surgery of Tongji Hospital affiliated with Tongji Medical College of Huazhong University of Science and Technology	Retrospective	1 Jan 2020- 20 Feb 2020	25 hospitalized patients with laboratory confirmed COVID-19	Severe (respiratory distress with respiratory rate ≥30/min, or oxygen saturation ≤93% at rest, or oxygenation index ≤ 300 mmHg), or critical (respiratory failure, shock, or organ dysfunction)	Smoking	8.75 (1.21-63.43)	N/A	N/A	28.0%	55.6%	12.5%	51
Liang et al [29]	China 575 hospitals in 31 provincial administrative regions	Retrospective	21 Nov 2019- 31 Jan 2020	1,590 hospitalized patients with laboratory confirmed COVID-19	Admission to the intensive care unit (ICU), invasive ventilation, or death	Current/former and never	2.34 (1.36-4.02)	N/A	N/A	7.0%	13.7%	6.4%	49

Table A1 Summary of Studies													
**Author**	**Setting**	**Study type**	**Time of data collection**	**Population**	**Definition of disease progression**	**Smoking status**	**Odds of disease progression (95%CI)**		**Adjustment**	**Smoking prevalence**			**Patients' mean age**
							**Unadjusted**	**Adjusted**		**Overall**	**More severe group**	**Less severe group**	
Liu et al [30]	China Three tertiary hospitals in Wuhan	Retrospective	30 Dec 2019-15 Jan 2020	78 hospitalized patients with laboratory confirmed COVID-19	Respiratory distress, respiratory failure, required mechanical ventilation and ICU care, or death	History of smoking	12.19 (1.76-84.31)	14.29 (1.58-25.0)	Age, maximum temperature at admission, respiratory failure, severe illness, albumin, creatinine, procalcitonin, and C-reactive protein level	6.4%	27.3%	3.0%	38
Mo et al [32]	China Zhongnan hospital of Wuhan University	Retrospective	1 Jan 2020-5 Feb 2020	155 hospitalized patients with laboratory confirmed COVID-19	Did not improve after treatment, status changed to severe, or hospitalized > 10 days	Current smoking	1.68 (0.30-9.45)	N/A	N/A	3.9%	4.7%	2.9%	54
Morassi et al [33]	Italy Fondazione Poliambulanza Hospital, Brescia, Italy and Udine University Hospital, Udine	Case series	16 Mar 2020- 5 Apr 2020	6 hospitalized patients with laboratory confirmed COVID-19 who developed clinical and neuroimaging evidence of acute stroke	Death	Smoker	1.00 (0.03-40.28)	N/A	N/A	16.7%	20.0%	0.0%	69

Table A1 Summary of Studies													
**Author**	**Setting**	**Study type**	**Time of data collection**	**Population**	**Definition of disease progression**	**Smoking status**	**Odds of disease progression (95%CI)**		**Adjustment**	**Smoking prevalence**			**Patients' mean age**
							**Unadjusted**	**Adjusted**		**Overall**	**More severe group**	**Less severe group**	
Palaiodimos et al [34]	US Montefiore Medical Center, Bronx, New York	Retrospective	9 Mar 2020- 22 Mar 2020	200 hospitalized patients with laboratory confirmed COVID-19	Increasing oxygen requirement*** Intubation In-hospital mortality	Current/former smoker	Increasing oxygen requirment: 1.86 (1.02-3.39) Intubation: 1.56 (0.77–3.15) In-hospital mortality: 1.19 (0.60-2.36)	Increasing oxygen requirement: 2.1 (1.07-4.1) Intubation: 1.66 (0.76–3.62) In-hospital mortality: 0.83 (0.37-1.87)	N/A	32.5%	N/A	N/A	64
Petrilli et al [35]	US NYU Langone Health with more than 260 outpatient office sites and four acute care hospitals in New York	Prospective cohort	1 Mar 2020- 8 Apr 2020	5,279 patients with laboratory confirmed COVID-19	Admission to intensive care unit, use of mechanical ventilation, discharge to hospice, or death	Current, former and never	Current smoker: 0.94 (0.65-1.36) Former smoker: 1.49 (1.22-1.82) Current/former: 1.37 (1.14-1.65)	Current smoker: 0.77 (0.52-1.20) Former smoker: 1.06 (0.85-1.30)	Age, sex, race, BMI, coronary arterial disease, heart failure, hyperlipidemia, hypertension, diabetes, asthma, COPD, chronic kidney disease, cancer, week of admission	Current smoker: 5.2% Former smoker: 20.6% Current/former: 25.7%	Current smoker: 4.4% Former smoker: 23.8% Current/former: 28.3%	Current smoker: 5.6% Former smoker: 18.7% Current/former: 24.3%	63
Qin et al [36]	China Tongji Hospital	Retrospective	10 Jan 2020- 12 Feb 2020	452 hospitalized patients with laboratory confirmed COVID-19	Respiratory distress with respiratory rate ≥30/min, or oxygen saturation ≤93% at rest, or oxygenation index ≤ 300 mmHg	Smoking	0.43 (0.10-1.94)	N/A	N/A	1.5%	1.0%	2.4%	58

Table A1 Summary of Studies													
**Author**	**Setting**	**Study type**	**Time of data collection**	**Population**	**Definition of disease progression**	**Smoking status**	**Odds of disease progression (95%CI)**		**Adjustment**	**Smoking prevalence**			**Patients' mean age**
							**Unadjusted**	**Adjusted**		**Overall**	**More severe group**	**Less severe group**	
Rastrelli et al [37]	Italy Carlo Poma Hospital in Mantua	Case series	No informatio n	31 male patients with laboratory confirmed COVID-19 in the respiratory intensive care unit	Transferred to ICU for intubation, or death	Current and former smoker	Current smoker: 2.33 (0.07-74.55) Former smoker: 1.89 (0.23-15.74) Current/former: 1.70 (0.21-14.02)	N/A	N/A	Current smoker: 3.2% Former smoker: 35.5% Current/f ormer: 38.7%	Current smoker : 0% Former smoker : 50% Current /former : 50%	Current smoker : 4.8% Former smoker : 42.9% Current /former : 47.7%	65
Ren et al [38]	Third People's Hospital of Shenzhen	Retrospective	11 Jan 2020- 12 Feb 2020	150 hospitalized patients with laboratory confirmed COVID-19	Development of sepsis	Smoking history	15.28 (0.77-301.86)	N/A	N/A	2.0%	6.1%	0.0%	54
Shi et al [39]	China Zhejiang Province	Retrospective	Up to 02/17/202 0	487 hospitalized patients with laboratory confirmed COVID-19	Severe disease (without precisely defining it) or death	Smoking history	1.61 (0.64-4.04)	N/A	N/A	8.2%	12.2%	7.8%	46
Wan et al [40]	China Chongqing University Three Gorges hospital, in northeast Chongqing	Retrospective	23 Jan 2020-8 Feb 2020	135 hospitalized patients with laboratory confirmed COVID-19	Respiratory distress with respiratory rate ≥30/min, or oxygen saturation ≤93% at rest, or oxygenation index ≤ 300 mmHg	Current smoking	0.28 (0.03-2.31)	N/A	N/A	6.7%	2.5%	8.4%	47
Wang et al [41]	NO.2 People's Hospital of Fuyang City	Retrospective	20 Jan 2020-9 Feb 2020	125 hospitalized patients with laboratory confirmed COVID-19	Severe (respiratory distress with respiratory rate ≥30/min, or oxygen saturation ≤93% at rest, or oxygenation index ≤ 300 mmHg), or critical (respiratory failure, shock, or organ dysfunction)	Current smoking	3.93 (1.30-11.93)	N/A	N/A	12.8%	28.0%	9.0%	39

Table A1 Summary of Studies

Author	Setting	Study type	Time of data collection	Population	Definition of disease progression	Smoking status	Odds of disease progression (95%CI)		Adjustment	Smoking prevalence			Patients' mean age
							Unadjusted	Adjusted		Overall	More severe group	Less severe group	
Xiong et al [42]	China Wuhan Hemodialysis Quality Control Center	Retrospective	1 Jan 2020- 10 Mar 2020	131 hospitalized patients with laboratory confirmed COVID-19 who received maintenance hemodialysis at 65 centers inWuhan, China	Severe (respiratory distress with respiratory rate ≥30/min, or oxygen saturation ≤93% at rest, or oxygenation index ≤ 300 mmHg), or critical (respiratory failure, shock, or organ dysfunction)	Current smoker	0.66 (0.26-1.69)	N/A	N/A	29.8%	23.3%	31.7%	63
Xu et al [43]	China First Affiliated Hospital, School of Medicine, Zhejiang University and Shenzhen Third People's Hospital	Retrospective	13 Jan 2020- 19 Feb 2020	113 hospitalized patients with laboratory confirmed COVID-19	Prolonged viral RNA shedding	Current smoker	0.80 (0.18-3.54)	N/A	N/A	7.1%	6.6%	8.1%	52
Yan et al [44]	China No.3 People's Hospital of Hubei Province	Retrospective	31 Jan 2020- 9 Mar 2020	168 hospitalized patients with laboratory confirmed COVID-19	Prolonged viral RNA shedding	Current smoker	0.86 (0.23-3.18)	N/A	N/A	10.0%	11.9%	8.2%	52
Yang et al [45]	China Wuhan Jin Yin-tan Hospital, Wuhan	Retrospective	24 Dec 2019-26 Jan 2020	52 critically ill hospitalized patients with laboratory confirmed COVID-19	Death	Smoking	0.11 (0.01-2.50)	N/A	N/A	3.8%	0.0%	10.0%	60

Table A1 Summary of Studies														
**Author**	**Setting**	**Study type**	**Time of data collection**	**Population**	**Definition of disease progression**	**Smoking status**	**Odds of disease progression (95%CI)**		**Adjustment**	**Smoking prevalence**			**Patients' mean age**
							**Unadjusted**	**Adjusted**		**Overall**	**More severe group**	**Less severe group**	
Yao et al [46]	China Dabieshan Medical Center 19 in Huanggang city, Hubei Province	Retrospective	30 Jan 2020-11 Feb 2020	108 hospitalized patients with laboratory confirmed COVID-19	Respiratory distress with respiratory rate ≥30/min, or breaths per minute, PaO2/FIO2 ratio lower than 250, multilobar infiltrates confusion, or disorientation, or respiratory failure, or required mechanical ventilation, or shock, or organ dysfunction, or death	Current smoker	11.18 (1.11-112.83)	N/A	N/A	3.7%	12.0%	1.2%	52
Yu Q et al [47]	24 designated hospitals in Jiangsu province	Retrospective	10 Jan 2020- 18 Feb 2020	421 hospitalized patients with laboratory confirmed COVID-19	Admission to ICU, acute respiratory failure occurrence, or shock	Current smoker	0.20 (0.01-3.37)	N/A	N/A	3.1%	0.0%	3.6%	47
Yu T et al [48]	China Dongguan People's Hospital and Nanfang Hospital, Southern Medical University	Retrospective	Jan 2020-Feb 2020	95 hospitalized patients with laboratory confirmed COVID-19	Pneumonia exacerbation	Tobacco smoking	6.67 (1.11-40.04)	16.13 (1.28-204.16)	Age, sex, blood pressure, BMI, serum lactic acid, neuotrophil count, lymphocyte count, hemoglobin, platelet count, creatinine, ALT, AST, and LDH	7.1%	21.1%	2.0%	43

Table A1 Summary of Studies													
**Author**	**Setting**	**Study type**	**Time of data collection**	**Population**	**Definition of disease progression**	**Smoking status**	**Odds of disease progression (95%CI)**		**Adjustment**	**Smoking prevalence**			**Patients' mean age**
							**Unadjusted**	**Adjusted**		**Overall**	**More severe group**	**Less severe group**	
Yu X et al [49]	Shanghai	Retrospective	1 Jan 2020- 19 Feb 2020	323 hospitalized patients with laboratory confirmed COVID-19	Severe (dyspnoea, and respiratory rate ≥ 30/min, blood oxygen saturation ≤ 93%, PaO2/ FiO2 ratio ≤ 300 mmHg); Critical (respiratory failure, or septic shock, or multiple organ dysfunction/ failure)	Smoking history	0.89 (0.20-4.01)	N/A	N/A	8.1%	8.0%	8.9%	50
Zhang J et al [50]	China No.7 Hospital of Wuhan	Retrospective	16 Jan 2020-3 Feb 2020	140 hospitalized patients with laboratory confirmed COVID-19	Respiratory distress with respiratory rate ≥30/min, or oxygen saturation ≤93% at rest, or oxygenation index ≤ 300 mmHg	Current and past smoker	Current smoker: 7.57 (0.35-160.88) Former smoker: 2.03 (0.44-9.42) Current/former: 3.04 (0.73-12.69)	N/A	N/A	Current smoker: 1.4% Former smoker: 5% Current/former: 6.4%	Current smoker : 3.4% Former smoker : 6.9% Current /former : 10.3%	Current smoker : 0% Former smoker : 3.7% Current /former : 3.7%	57
Zhang R et al [51]	China Renmin Hospital of Wuhan University	Retrospective	10 Jan 2020- 10 Feb 2020	120 hospitalized patients with laboratory confirmed COVID-19	Respiratory distress with respiratory rate ≥30/min, or oxygen saturation ≤93% at rest, or oxygenation index ≤ 300 mmHg, or respiratory failure requiring mechanical ventilation, shock, or organ dysfunction requiring ICU treatment	Current smoking	48.02 (2.61-882.27)	N/A	N/A	5.0%	20.0%	0.0%	45
Zhang X et al [52]	China Zhejiang Province	Retrospective	17 Jan 2020-8 Feb 2020	645 patients confirmed with SARSCoV-2 infection	Abnormal imaging findings (CT scan or Chest X-ray)	Current smoker	1.17 (0.41-3.39)	N/A	N/A	6.4%	6.5%	5.6%	47

Table A1 Summary of Studies

Author	Setting	Study type	Time of data collection	Population	Definition of disease progression	Smoking status	Odds of disease progression (95%CI)		Adjustment	Smoking prevalence			Patients' mean age
							Unadjusted	Adjusted		Overall	More severe group	Less severe group	
Zheng Y et al [53]	China Taihe Hospital at Shiyan	Retrospective	16 Jan 2020- 4 Feb 2020	73 hospitalized patients with laboratory confirmed COVID-19	Severe (dyspnoea, and respiratory rate ≥ 30/min, blood oxygen saturation ≤ 93%, PaO2/ FiO2 ratio ≤ 300 mmHg); Critical (respiratory failure, or septic shock, or multiple organ dysfunction/ failure)	Smoker	0.44 (0.08-2.35)	N/A	N/A	11.0%	6.7%	14.3%	43
Zhou et al [54]	China Jin Yin-tan Hospital and Wuhan Pulmonary Hospital in Wuhan	Retrospective	29 Dec 2019-31 Jan 2020	191 hospitalized patients with laboratory confirmed COVID-19	Death	Current smoker	2.23 (0.65-7.63)	N/A	N/A	5.8%	4.4%	9.3%	56

* Hospitalization status unknown was excluded from the analysis.
** Two children were excluded from the analysis.
*** Increasing oxygen requirement was used in the analysis.

**Table A2 Risk of Bias of Studies** (2=high risk, 1=intermediate risk, 0=low risk)						
**Study**	**Study populatio n**	**Exposure measurement**	**Outcome assessment**	**Measurement of confounders**	**Adequate follow-up**	**Average score**
Beyrouti et al [8]	2	2	0	2	0	1.2
Buckner et al [9]	0	2	0	2	0	0.8
CDC COVID-19 [10]	0	0	0	2	0	0.4
Chen Q et al [11]	0	2	0	2	0	0.8
Chen T et al [12]	0	0	0	2	0	0.4
Dong et al [13]	2	2	2	2	0	1.6
Feng et al [14]	0	2	0	2	0	0.8
Gayam et al [15]	2	2	0	2	0	1.2
Giacomelli et al [16]	0	2	0	2	0	0.8
Goyal et al [17]	0	2	0	2	0	0.8
Guan et al [18]	0	0	0	2	0	0.4
Guo et al [19]	0	2	0	2	0	0.8
Hu et al [20]	0	2	0	0	0	0.4
Huang C et al [21]	1	2	0	2	0	1
Huang R et al [22]	0	2	0	2	0	0.8
Hur et al [23]	0	1	0	2	0	0.6
Ji Dong et al [24]	0	2	0	2	0	0.8
Kim et al [25]	1	2	0	2	0	1
Klang et al [26]	0	2	0	0	0	0.4
Li X et al [27]	0	0	0	2	0	0.4
Li YK et al [28]	1	2	0	2	0	1
Liang et al [29]	0	1	0	2	0	0.6
Liu et al [30]	0	2	0	0	0	0.4
Mo et al [32]	0	2	0	2	0	0.8
Morassi et al [33]	2	2	0	2	0	1.2
Palaiodimos et al [34]	0	1	0	0	0	0.2
Petrilli et al [35]	0	0	0	0	0	0
Qin et al [36]	0	2	0	2	0	0.8
Rastrelli et al [37]	1	0	0	2	2	1
Ren et al [38]	0	2	0	2	0	0.8
Shi et al [39]	0	2	0	2	2	1.2
Wan et al [40]	0	2	0	2	0	0.8
Wang et al [41]	0	2	0	2	0	0.8
Xiong et al [42]	0	2	0	2	0	0.8
Xu et al [43]	0	2	0	2	0	0.8
Yan et al [44]	0	2	0	2	0	0.8

**Table A2 Risk of Bias of Studies** (2=high risk, 1=intermediate risk, 0=low risk)						
**Study**	**Study populatio n**	**Exposure measurement**	**Outcome assessment**	**Measurement of confounders**	**Adequate follow-up**	**Average score**
Yang et al [45]	0	2	0	2	0	0.8
Yao et al [46]	0	2	0	2	0	0.8
Yu Q et al [47]	0	2	0	2	0	0.8
Yu T et al [48]	0	2	0	0	2	0.8
Yu X et al [49]	0	2	0	2	0	0.8
Zhang J et al [50]	0	0	0	2	0	0.4
Zhang R et al [51]	0	2	0	2	0	0.8
Zhang X et al [52]	0	2	0	2	0	0.8
Zheng Y et al [53]	0	2	0	2	0	0.8
Zhou et al [54]	0	2	0	2	0	0.8

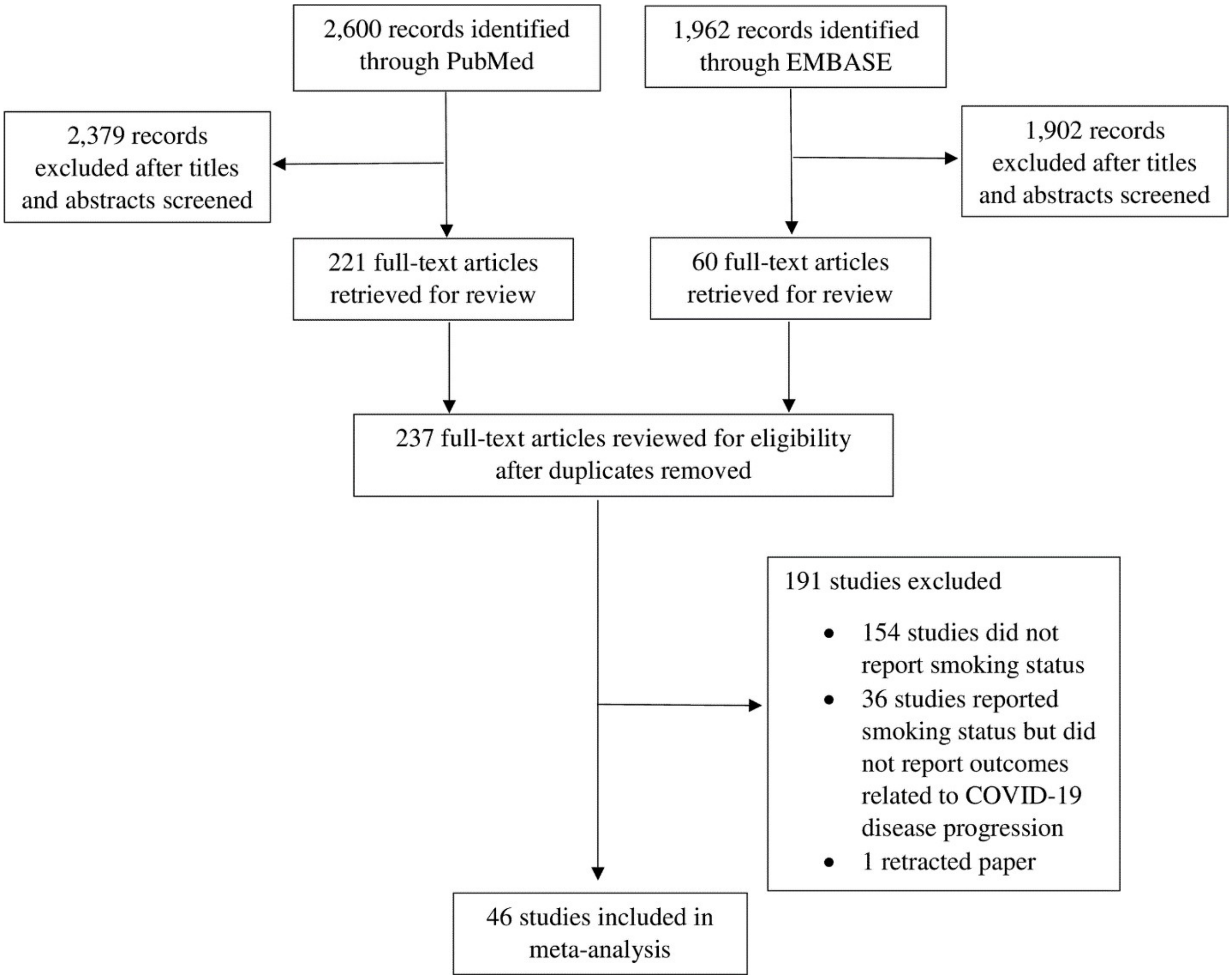

Figure A1. PRISMA diagram

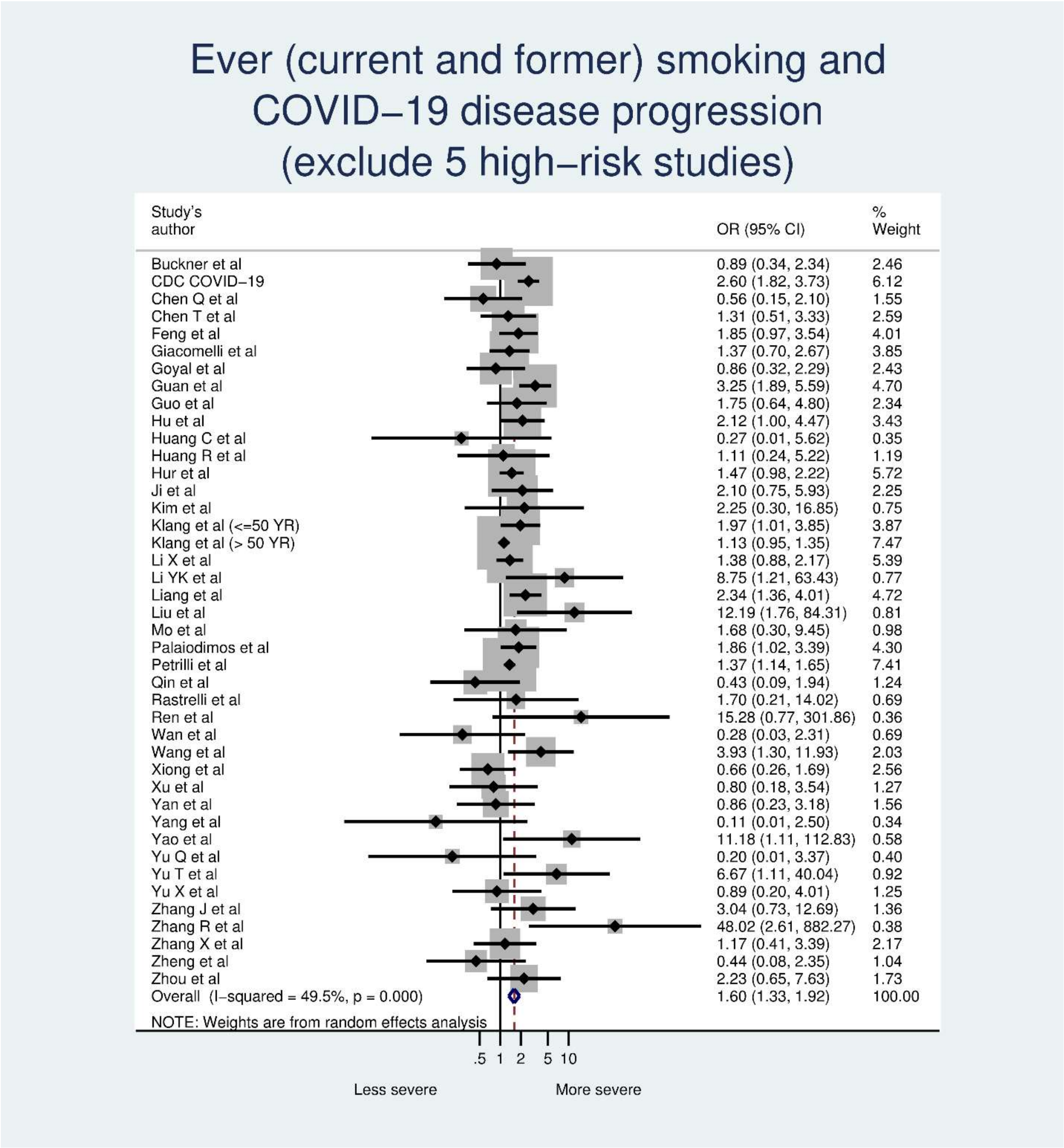

Figure A2. Ever (current and former) smoking and COVID-19 disease progression, dropping 5 studies with high risk of bias scores.

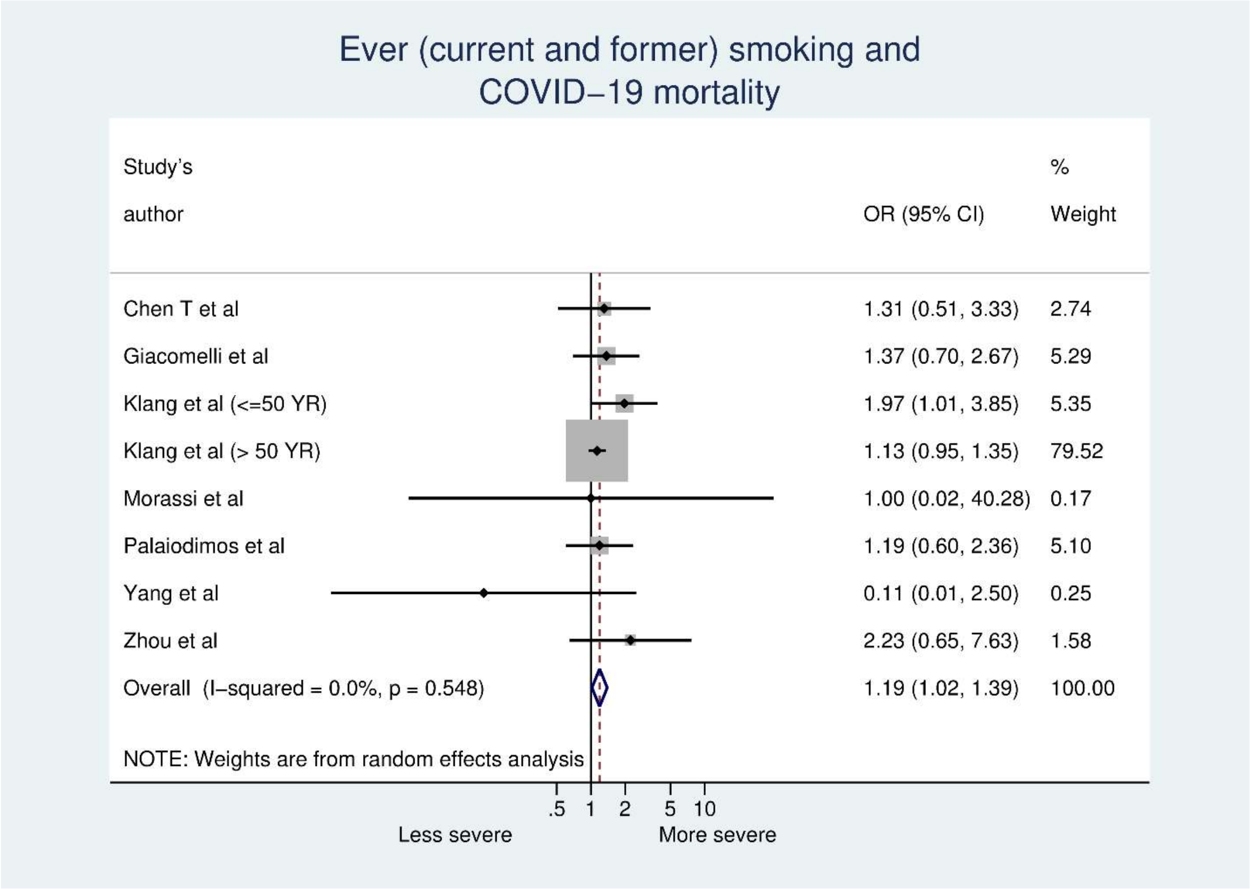

Figure A3. Ever (current and former) smoking and COVID-19 mortality

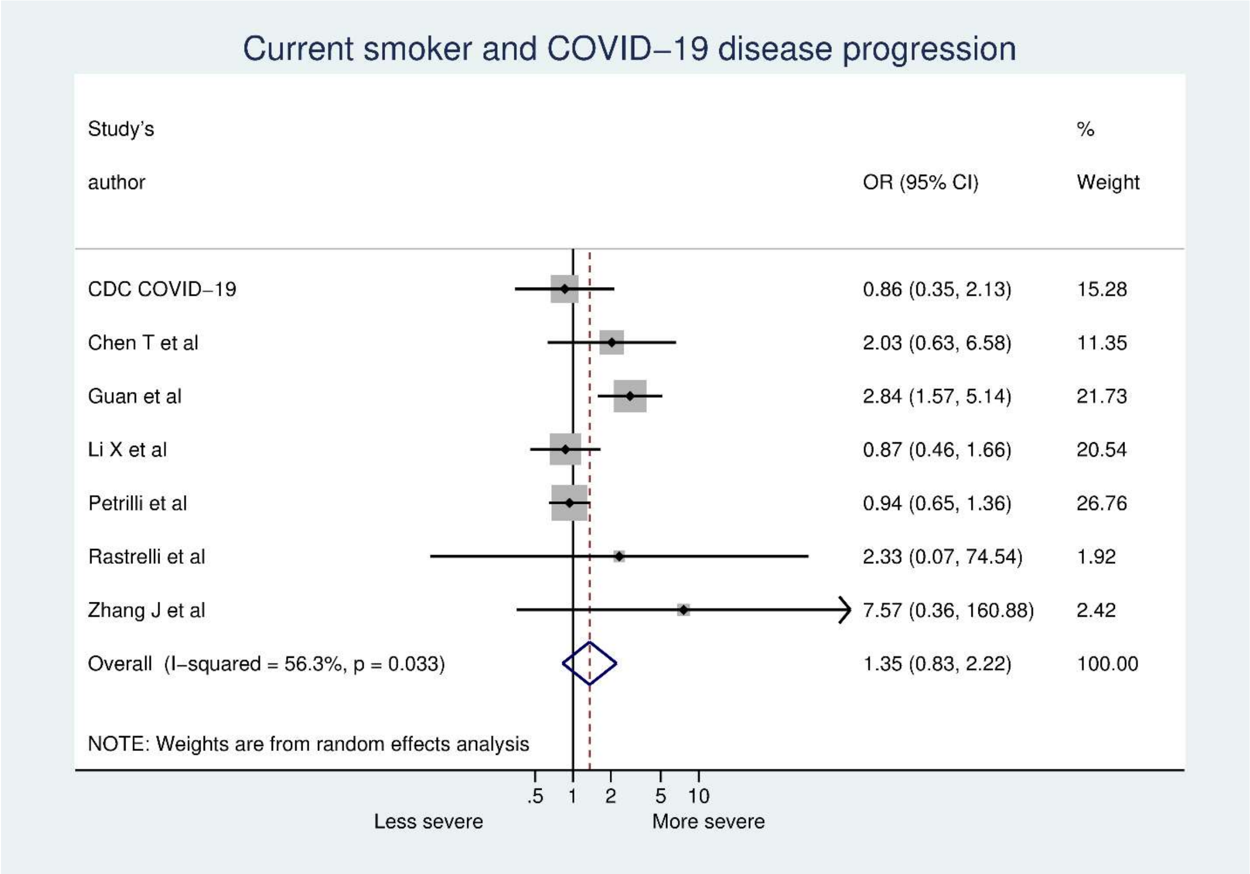

Figure A4. Current smokers vs. never smokers and COVID-19 disease progression.

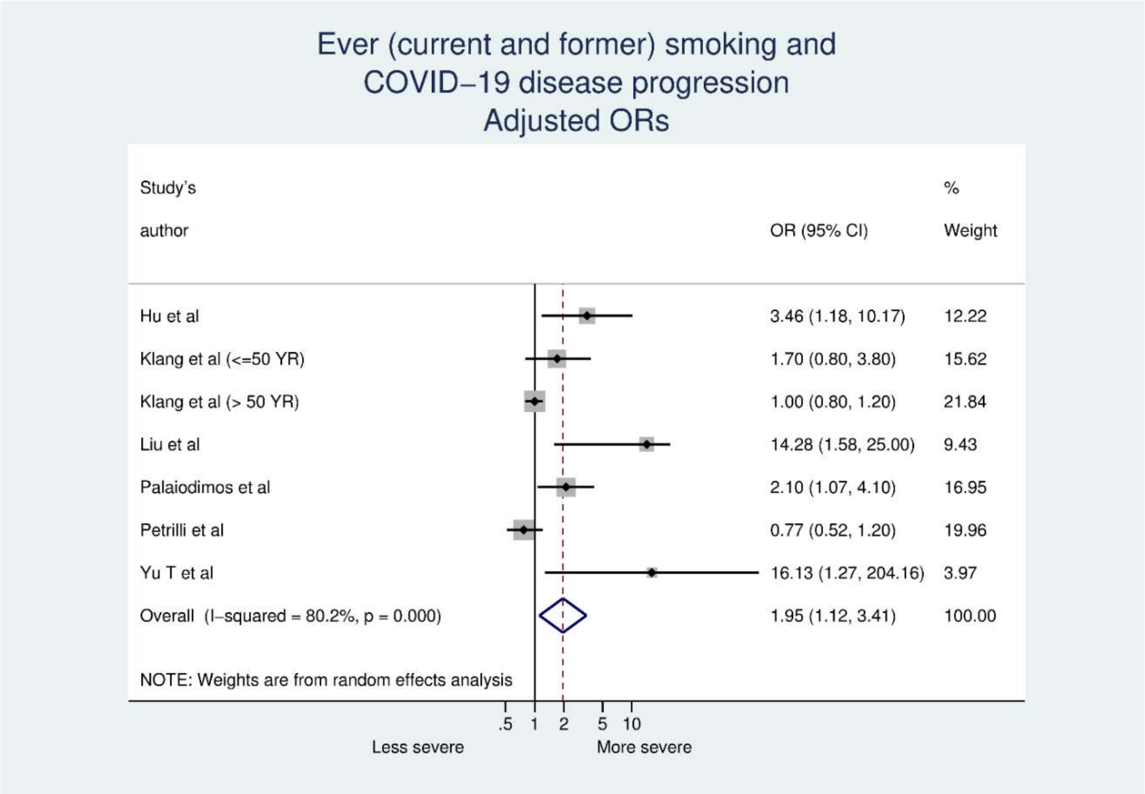

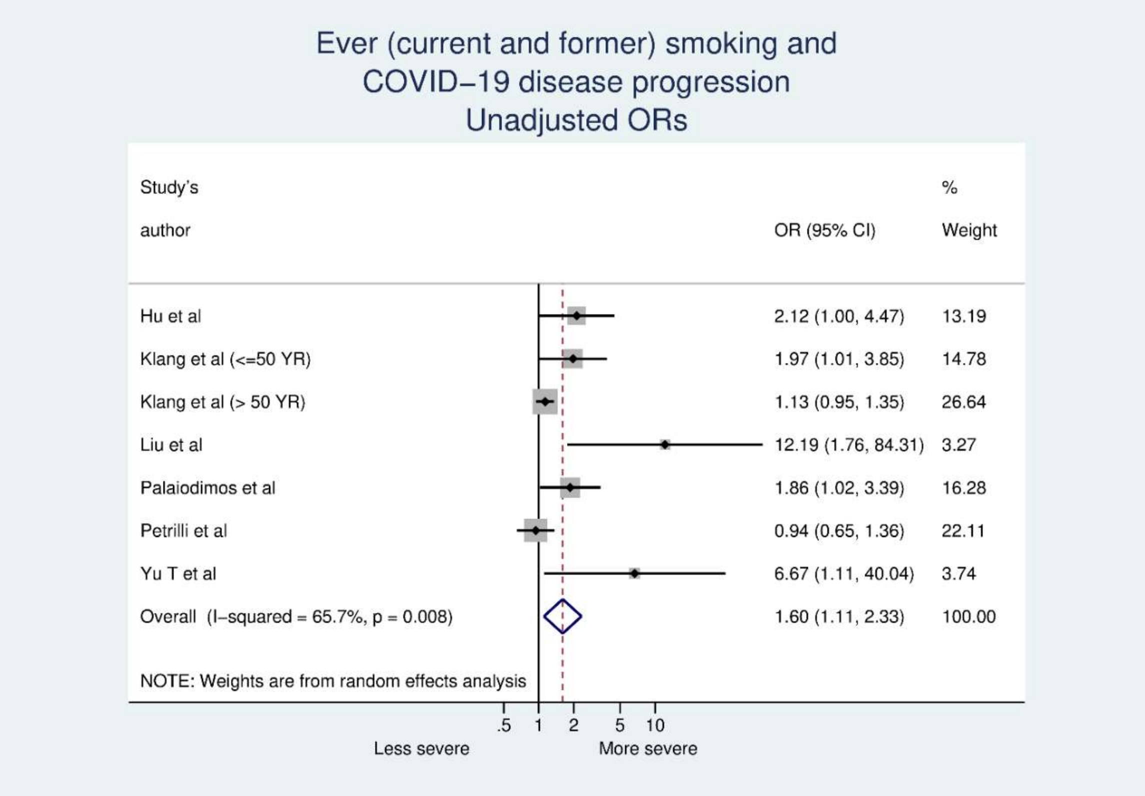

Figure A5. Ever (current and former) smoking and disease progression in models that adjusted (top) and did not adjust (bottom) ORs for confounding variables.
